# Chromatin Remodeling, Cell Proliferation and Cell Death in Valproic Acid-Treated HeLa Cells

**DOI:** 10.1371/journal.pone.0029144

**Published:** 2011-12-19

**Authors:** Marina Barreto Felisbino, Wirla M. S. C. Tamashiro, Maria Luiza S. Mello

**Affiliations:** 1 Department of Structural and Physiological Biology, Institute of Biology, University of Campinas (UNICAMP), Campinas, São Paulo, Brazil; 2 Department of Genetics, Evolution and Bioagents, Institute of Biology, University of Campinas (UNICAMP), Campinas, São Paulo, Brazil; The University of Kansas Medical Center, United States of America

## Abstract

**Background:**

Valproic acid (VPA) is a potent anticonvulsant that inhibits histone deacetylases. Because of this inhibitory action, we investigated whether VPA would affect chromatin supraorganization, mitotic indices and the frequency of chromosome abnormalities and cell death in HeLa cells.

**Methodology/Principal Findings:**

Image analysis was performed by scanning microspectrophotometry for cells cultivated for 24 h, treated with 0.05, 0.5 or 1.0 mM VPA for 1–24 h, and subjected to the Feulgen reaction. TSA-treated cells were used as a predictable positive control. DNA fragmentation was investigated with the TUNEL assay. Chromatin decondensation was demonstrated under TSA and all VPA treatments, but no changes in chromosome abnormalities, mitotic indices or morphologically identified cell death were found with the VPA treatment conditions mentioned above, although decreased mitotic indices were detected under higher VPA concentration and longer exposure time. The frequency of DNA fragmentation identified with the TUNEL assay in HeLa cells increased after a 24-h VPA treatment, although this fragmentation occurred much earlier after treatment with TSA.

**Conclusions/Significance:**

The inhibition of histone deacetylases by VPA induces chromatin remodeling in HeLa cells, which suggests an association to altered gene expression. Under VPA doses close to the therapeutic antiepileptic plasma range no changes in cell proliferation or chromosome abnormalities are elicited. The DNA fragmentation results indicate that a longer exposure to VPA or a higher VPA concentration is required for the induction of cell death.

## Introduction

Valproic acid (VPA) is a drug widely prescribed for the treatment of seizure disorders, including epilepsy, episodes related to bipolar disorder and migraine headaches [1]–[3]. In addition to inhibiting the transamination of the neurotransmitter GABA and blocking the voltage-gated sodium channels and T-type calcium channels [4] within a therapeutic antiepileptic plasma range (0.3 mM-0.7 mM), VPA is a potent inhibitor of class I histone deacetylases (HDACs) in several cell types [1], [5]–[12]. Recent findings have supported the proposal of a dynamic interplay between histone acetylation, histone methylation and DNA demethylation in response to VPA treatment in certain cell systems [7], [13]–[15].

While there is only a minute amount of acetylated histones in HeLa cells, treatment of these cells with VPA at a concentration as low as 0.25 mM increases the amount of acetylated histone H4, and treatment at a concentration of 2 mM induces its massive acetylation [5]. Accumulation of acetylated histone H4 was observed as early as 1 h after the addition of 0.5 or 1.0 mM VPA to the culture medium [6]. Maximum histone H4 acetylation in HeLa cells appears nearly 12-16 h after VPA addition [6]. Acetylation of histone H3 also significantly increases in HeLa and L929 cells after VPA treatment [16], [17].

The epigenetic effect of hyperacetylation of histones by VPA activates transcription from diverse promoters [1] and has been considered promising for the control of certain cell malignancies [5], [8], [18]–[21]. On the other hand, the hyperacetylation of histones induced by a class I-specific HDAC inhibitor (HDACi) like VPA or by a pan-HDACi such as trichostatin A (TSA) [11] has been considered totally or at least partly responsible for the similar teratogenic side-effects in vertebrate embryos [1], [17], [22].

Changes in chromatin supraorganization and nuclear architecture in HeLa cells after treatment with TSA have been described as a result of the enhanced acetylation of nucleosome core histones [23], [24]. Exposure to VPA, in addition to histone acetylation, has also been shown to induce the depletion of proteins that maintain chromatin structure in breast MCF-7 cancer cells thus leading to the potentiation of DNA-damaging agents [7]. Treatment of prostate cancer cells *in vitro* and *in vivo* with VPA has been reported to result in dose- and time-dependent changes in nuclear structure [8].

HDACis have also been implicated in the cell cycle arrest and apoptosis intrinsic and extrinsic pathways, inducing the expression of genes encoding cell death receptors and their cognate ligands or mediating the repression of genes that encode inhibitors of these pathways [18], [25]–[33]. In addition to eliciting apoptotic pathways, HDACis have been reported to be involved with the induction of autophagic cell death, mitotic cell death and senescence in various transformed cell lines [27], [33]. Acetylation of non-histone proteins by HDACis may account for their reported antitumor responses or synergistic effects when HDACis are combined with pro-apoptotic agents [26], [27], [31].

Here, we investigated whether VPA, as a consequence of its epigenetic action, would affect chromatin supraorganization in HeLa cells similarly to TSA in these cells [23] or to VPA in prostate cancer cells [8] or even in breast cancer cells [7], using an appropriate image analysis method. In addition, as cell proliferation and cell death may also be affected by VPA, Feulgen-DNA C classes, cell death morphological aspects and DNA fragmentation were investigated in parallel with chromatin texture analysis and with the frequency of chromosome abnormalities.

## Results

### Image Analysis

**Figure 1:**
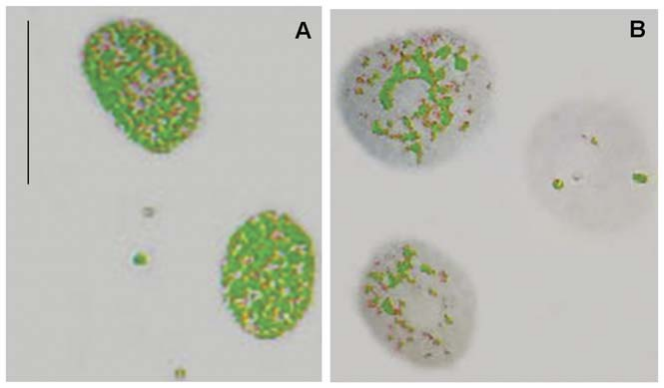
False-colored images of Feulgen-stained HeLa cells. Condensed chromatin packaging revealed as green points is less frequent in VPA-treated cells (B) in comparison with untreated controls (A). Scale bar, 25 µm.

**Figure 2:**
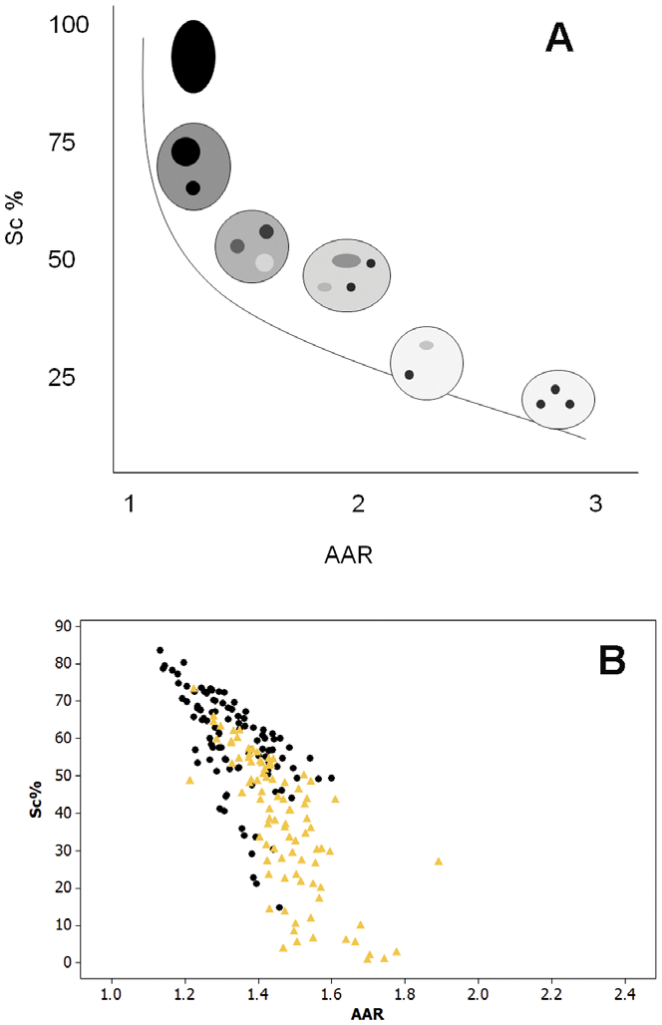
Scatter diagrams representing positioning of nuclei with different phenotypic images in Feulgen-stained cells. These diagrams relate Sc% (relative condensed chromatin area) and AAR (the level of textural contrast between condensed and non-condensed chromatin). A. Schematic model diagram modified from Vidal (1984) and Mello et al. (2009). B. HeLa cells treated with 100 ng/mL TSA (yellow), in comparison to untreated controls (black).

False-color images suggestive of chromatin unpackaging could be observed especially with shorter (1-2 h) VPA treatment in the Feulgen-stained nuclei. Nuclear areas covered by condensed chromatin packaging (revealed as green points), which were abundant in untreated controls (Fig. 1 A), appeared reduced in VPA-treated cells (Fig. 1 B). Scatter diagrams in which nuclear relative areas covered with condensed chromatin (Sc %) were related to the level of textural contrast between condensed and non-condensed chromatin (AAR), were obtained for the nuclei analyzed by scanning microspectrophotometry. These scatter plots were compared with the schematic model by Vidal [34] and Mello et al. [35], which allows for the discrimination of the position in this diagram of points corresponding to specific nuclear phenotypic images (Fig. 2 A). When HeLa cells were treated with 100 ng/mL TSA as a positive control to induce chromatin loosening, decreased Sc% values with increasing AAR values were demonstrated in the scatter plot representation (Fig. 2 B) or after statistical comparison (Table 1).

**Table 1:** Condensed chromatin area and chromatin textural contrast in HeLa cells treated with 100 ng/mL TSA for 24 h.

Treatment	Sc %			AAR		
	X	S	Md	X	S	Md
Untreated control	58.77	13.12	60.05^a^	1.33	0.10	1.32^a^
TSA	37.74	18.20	40.86^b^	1.47	0.12	1.45^b^

a,b , The median values of the TSA and control groups differ significantly from each other at the P_0.05_ level (Mann-Whitney test); AAR, average absorption ratio; Md, median: S, standard deviation; Sc %, relative condensed chromatin area; TSA, trichostatin A; X, arithmetic mean; n, 100

**Figure 3:**
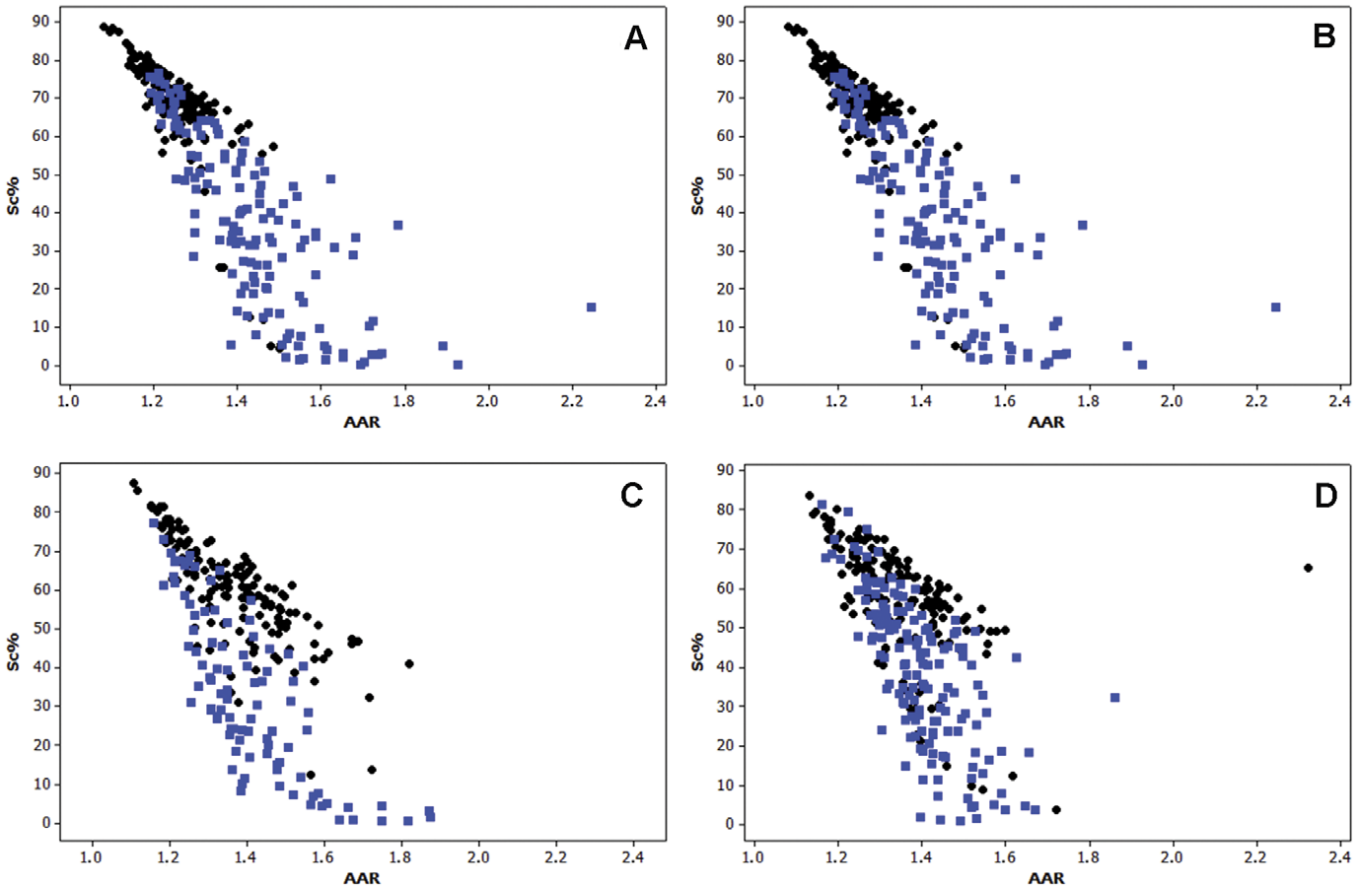
Scatter diagrams of Sc% vs. AAR for Feulgen-stained HeLa cells. The nuclei from cells treated with 0.05 mM VPA for 1 (A), 2 (B), 4 (C), or 24 h (D) are represented in blue in comparison with untreated controls, which are represented in black. n, 200.

**Figure 4:**
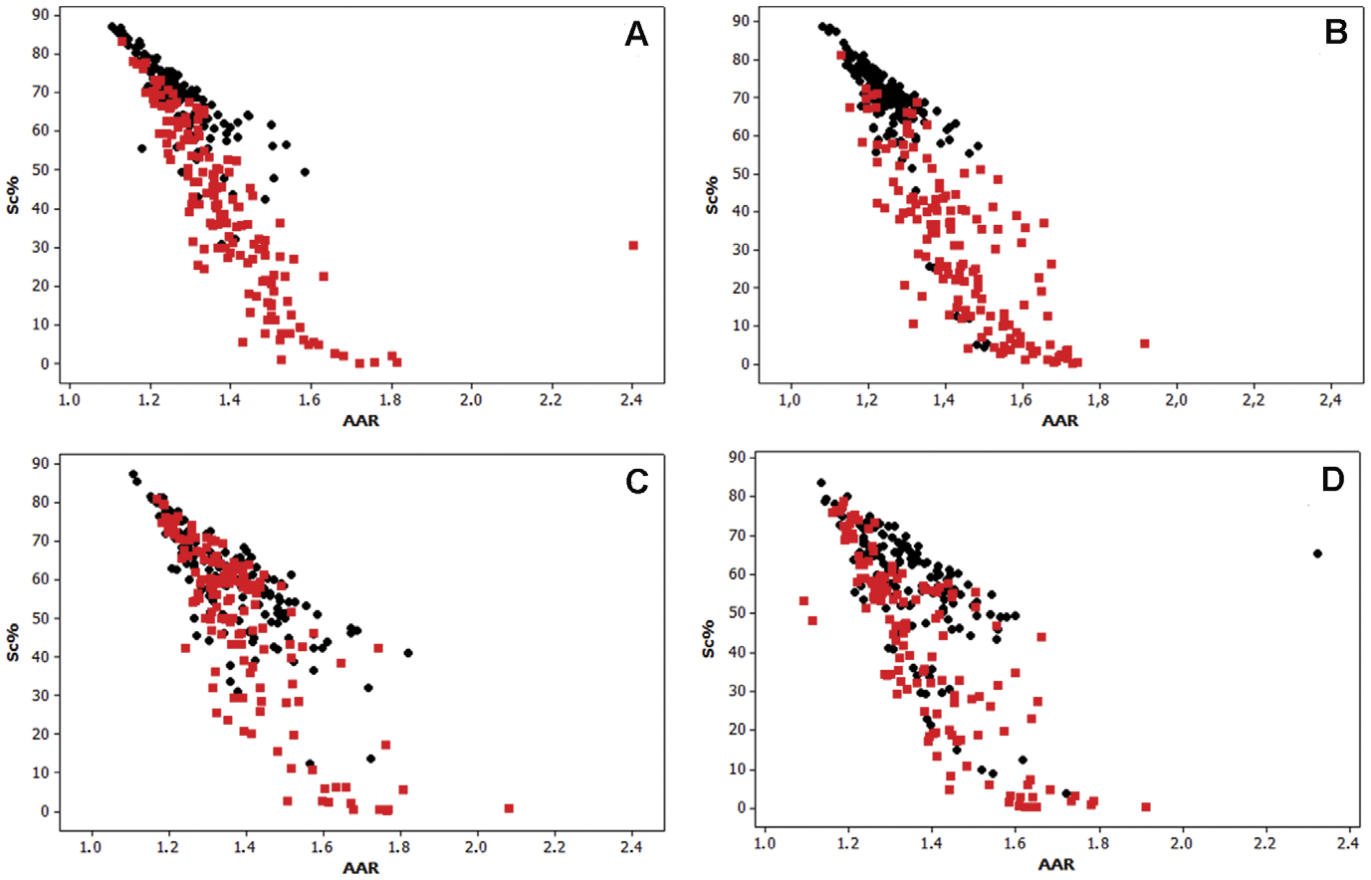
Scatter diagrams of Sc% vs. AAR for Feulgen-stained HeLa cells. The nuclei from cells treated with 0.5 mM VPA for 1 (A), 2 (B), 4 (C), or 24 h (D) are represented in red in comparison with untreated controls, which are represented in black. n, 200.

**Figure 5:**
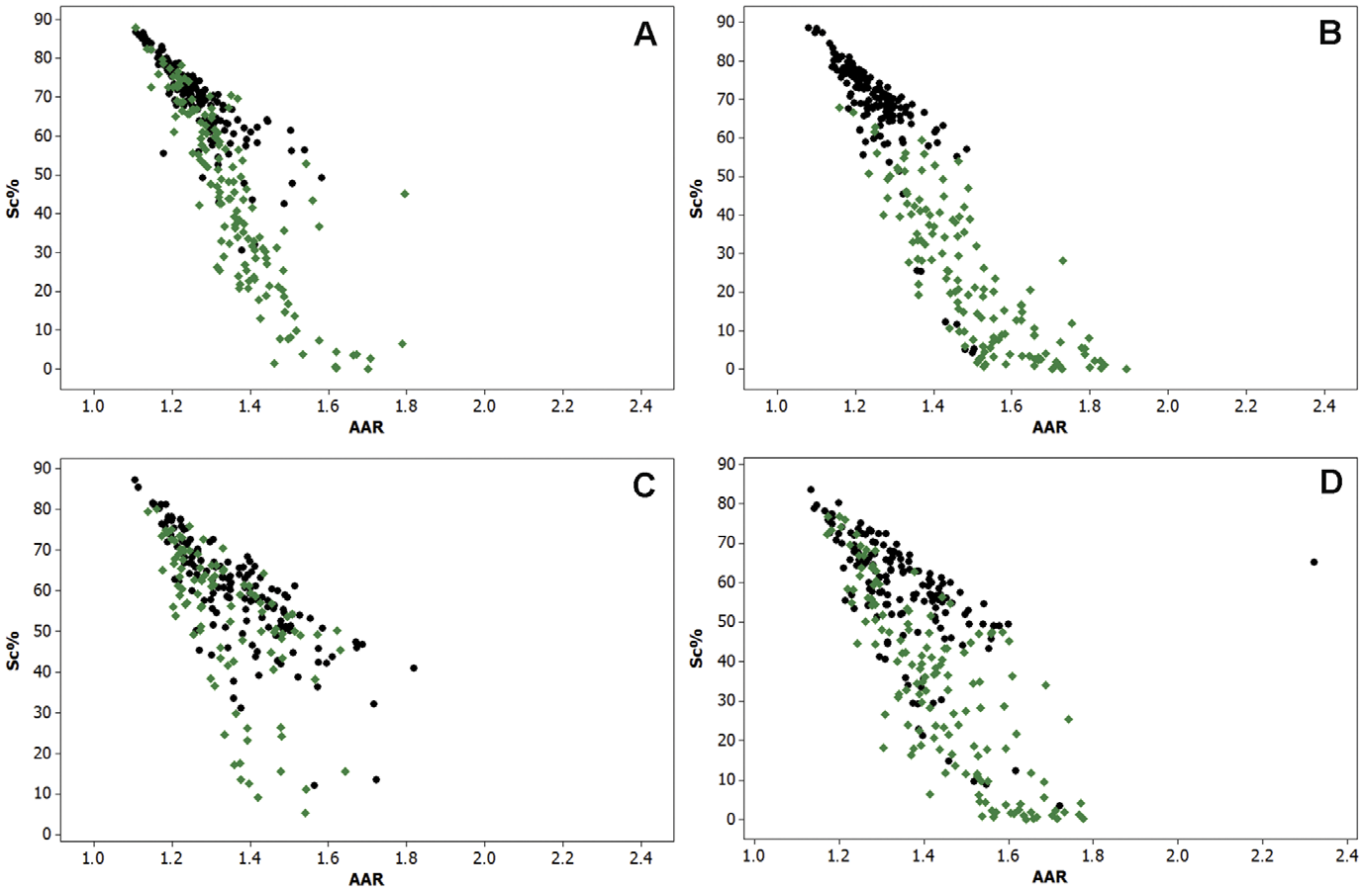
Scatter diagrams of Sc% vs. AAR for Feulgen-stained HeLa cells. The nuclei from cells treated with 1.0 mM VPA for 1 (A), 2 (B), 4 (C), or 24 h (D) are represented in green in comparison with untreated controls, which are represented in black. n, 200.

Similar to the data obtained for TSA-treated cells, the results obtained for the cells treated with 0.05, 0.5 or 1.0 mM VPA for 1, 2, 4 or 24 h showed decreasing values of Sc % and generally increased values of AAR in comparison with the respective untreated controls (Fig. 3, 4, 5; Tables 2, 3, 4).

**Table 2:** Condensed chromatin area and chromatin textural contrast in 0.05 mM VPA- treated HeLa cells

Treatment		Sc%			AAR		
time (h)	Drug	X	S	Md	X	S	Md
1	Untreated control	69.17	10.13	70.77^a^	1.27	0.09	1.26^a^
	VPA	36.33	25.04	38.22^b^	1.40	0.17	1.37^b^
2	Untreated control	68.53	14.52	70.65^a^	1.25	0.08	1.24^a^
	VPA	37.29	22.07	36.85^b^	1.44	0.16	1.42^b^
4	Untreated control	60.15	13.17	60.86^a^	1.36	0.14	1.35^a^
	VPA	33.43	20.81	31.25^b^	1.40	0.15	1.38^b^
24	Untreated control	57.75	14.84	59.91^a^	1.35	0.14	1.32^a^
	VPA	38.76	18.96	40.84^b^	1.40	0.11	1.39^b^

a,b , The median values of the VPA group and the respective control for each treatment time differ significantly from each other at the P _0.05_ level (Mann-Whitney test); AAR, average absorption ratio; Md, median; S, standard deviation; Sc %, relative condensed chromatin area; VPA, valproic acid; X, arithmetic mean; n, 200

**Table 3:** Condensed chromatin area and chromatin textural contrast in 0.5 mM VPA- treated HeLa cells.

Treatment		Sc%			AAR		
time (h)	Drug	X	S	Md	X	S	Md
1	Untreated control	69.17	10.13	70.77^a^	1.27	0.09	1.26^a^
	VPA	41.30	21.91	41.76^b^	1.39	0.16	1.36^b^
2	Untreated control	68.53	14.52	70.65^a^	1.25	0.08	1.24^a^
	VPA	30.51	20.73	31.11^b^	1.44	0.15	1.43^b^
4	Untreated control	60.15	13.17	60.86^a^	1.36	0.14	1.35^a^
	VPA	49.52	21.42	56.41^b^	1.39	0.15	1.38^a^
24	Untreated control	57.75	14.84	59.91^a^	1.35	0.14	1.32^a^
	VPA	42.31	22.42	47.41^b^	1.38	0.15	1.34^a^

a,b , The median values of the VPA groups and their respective control for each treatment time differ significantly from each at the P _0.05_ level (Mann-Whitney test); AAR, average absorption ratio; Md, median; S, standard deviation; Sc %, relative condensed chromatin area; VPA, valproic acid; X, arithmetic mean; n, 200

**Table 4:** Condensed chromatin area and chromatin textural contrast in 1.0 mM VPA- treated HeLa cells.

Treatment		Sc%			AAR		
time (h)	Drug	X	S	Md	X	S	Md
1	Untreated control	69.17	10.13	70.77^a^	1.27	0.09	1.26^a^
	VPA	44.53	22.93	45.57^b^	1.36	0.13	1.34^b^
2	Untreated control	68.53	14.52	70.65^a^	1.25	0.08	1.24^a^
	VPA	23.96	18.88	20.67^b^	1.50	0.16	1.48^b^
4	Untreated control	60.15	13.17	60.86^a^	1.36	0.14	1.35^a^
	VPA	53.20	17.86	56.93^b^	1.34	0.12	1.32^a^
24	Untreated control	57.75	14.84	59.91^a^	1.35	0.14	1.32^a^
	VPA	34.18	22.18	35.55^b^	1.44	0.15	1.42^b^

a,b , The median values of the VPA groups and their respective control for each treatment time differ significantly at the P _0.05_ level (Mann-Whitney test); AAR, average absorption ratio; Md, median; S, standard deviation; Sc %, relative condensed chromatin area; VPA, valproic acid; X, arithmetic mean; n, 200

DNA content was not found to increase with VPA treatments. Indeed, the significant decrease in A_T_ values in the VPA-treated cells (Table 5) was due to a smaller frequency of nuclei with larger Feulgen-DNA amounts in comparison to respective controls (Fig. S1, S2, S3, and S4). An increase in the nuclear areas after 1- and 2-h VPA treatments was demonstrated statistically by the Mann-Whitney test (Table 5); however, after longer exposure times, this increase was masked when considering the arithmetic means and medians of the S_T_ values, most likely because the frequency of nuclei with the highest Feulgen-DNA values under these conditions diminished (Fig. S1, S2, S3, and S4).

**Table 5:** DNA content and nuclear area in VPA-treated HeLa cells

Treatment		A_T_ (arbitrary units)			S_T_ (µm^2^)		
time (h)	VPA (mM)	X	S	Md	X	S	Md
1	zero	123.91	55.28	100.25^a^	209.95	71.54	191.00^a^
	0.05	87.93	33.91	74.80^b^	242.21	61.76	231.25^b^
	0.5	88.45	34.01	79.32^b^	236.06	81.05	222.88^c^
	1.0	95.76	46.93	79.43^b^	237.78	84.88	216.25^c^
2	zero	138.86	62.13	116.72^a^	251.97	88.40	237.50^a^
	0.05	108.73	46.60	98.00^b^	311.12	110.77	296.88^b^
	0.5	99.77	41.56	95.56^b^	301.52	108.03	295.13^b^
	1.0	60.30	21.85	60.66^c^	199.95	57.02	193.00^c^
4	zero	143.62	85.52	115.56^a^	287.55	102.09	261.50^a^
	0.05	88.20	31.52	82.01^b^	262.89	95.41	242.88^b^
	0.5	109.32	52.79	96.44^c^	262.32	77.61	241.50^c^
	1.0	100.82	31.17	93.18^c^	248.93	77.17	236.00^b^
24	zero	138.97	72.65	112.06^a^	306.20	103.73	288.50^a^
	0.05	93.43	41.31	90.86^b^	263.90	104.72	261.00^b^
	0.5	98.31	40.05	94.42^b^	270.31	87.50	253.75^b^
	1.0	100.03	42.13	92.73^b^	292.64	90.93	277.75^a^

b, c , The median values of the VPA groups differ significantly from each other and from their respective control (zero,

a ) at the P _0.05_ level (Mann-Whitney test)

AT, nuclear DNA content; Md, median; S, standard deviation; S_T,_ nuclear area; VPA, valproic acid; n, 200 cells per experimental condition.

In most cases, > 50% of the nuclei were found to present a Feulgen-DNA content in the “∼2 C” Feulgen-DNA degree class (G1 phase) irrespective of control or VPA treatments (Figs. S1, S2, S3, and S4). The “∼2 C” degree class was named here as the class containing the smallest values presented by control cells after 1 h in growing conditions, considering that HeLa cells constitute a heteroploid cell strain with aneuploidy characteristics [36], and was also comparable to the Feulgen-DNA value distribution presented by human lymphocytes (Fig. S1). A decrease in the frequency of nuclei in the next doubling DNA C classes was generally induced by VPA as early as 1 h following treatment and at a drug dose as low as 0.05 mM. Conversely, an increase in the frequency of nuclei with Feulgen-DNA values < “∼2C” occurred with the VPA treatments.

All of these data indicate that chromatin decondensation occurred under the treatment conditions studied here.

### Mitotic Indices, Mitotic Abnormalities, Micronucleation and Cell Death Ratios

No statistically significant changes in the mitotic index were observed for the 0.05, 0.5 or 1.0 mM VPA treatments for 1, 2, 4 and 24 h (Table S1). However, when the cells were treated for 48 h at a VPA concentration that is considerably higher (5.0 mM) than the therapeutic range [5], [6], the mitotic indices significantly decreased (1).

**Figure 6:**
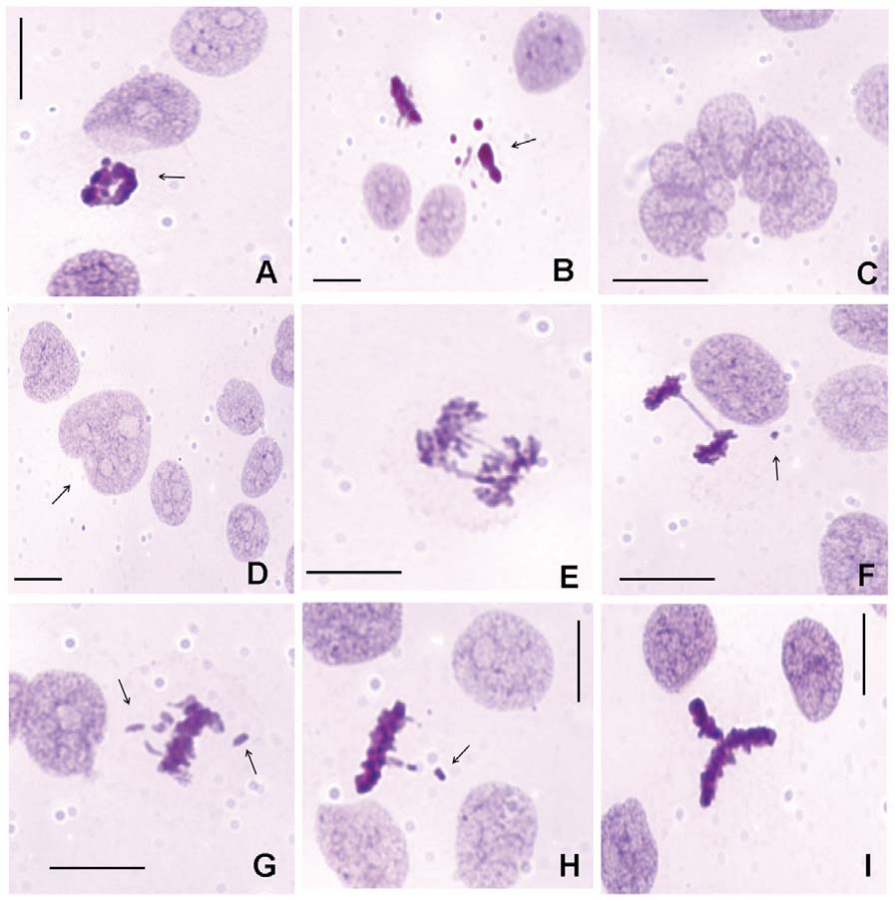
Cell death aspects, mitotic abnormalities and micronuclei in Feulgen-stained HeLa cells. Apoptosis (A, B – arrows), cell death preceded by multinucleation (C), a giant nucleus (D – arrow), chromosome bridges (E, F), a micronucleus (F – arrow), lagging chromosomes (G, H – arrows), and a tripolar spindle (I) are observed. Scale bar, 25 µm.

Abnormal mitoses were observed under all experimental conditions analyzed. However, no change in the frequency of mitotic abnormalities, micronuclei and giant nuclei was introduced by the VPA treatment, except for a decrease in the frequency of giant nuclei after treatment with 5.0 mM VPA for 48 h (Table S1). The high frequency of abnormal mitosis in the preparations treated with 5.0 mM VPA for 48 h resulted from the reduced number of mitoses under this condition and their generally abnormal characteristics of the cells (Table S1). Examples of mitotic abnormalities, such as tripolar spindles, chromosome bridges, lagging chromosomes, a micronucleated cell and a giant nucleus, are shown in Figures 6 D-I.

Cell death morphologies associated with apoptosis and cell death preceded by multinucleation (Fig. 6 A–C) were also observed in all cell preparations examined. The apoptotic ratios and the frequency of cell death preceded by multinucleation based on cell morphological aspects detected in the Feulgen-stained preparations were not affected by VPA treatments in comparison with untreated controls (Table S1).

### DNA Fragmentation as Assessed by the TUNEL Assay

**Figure 7:**
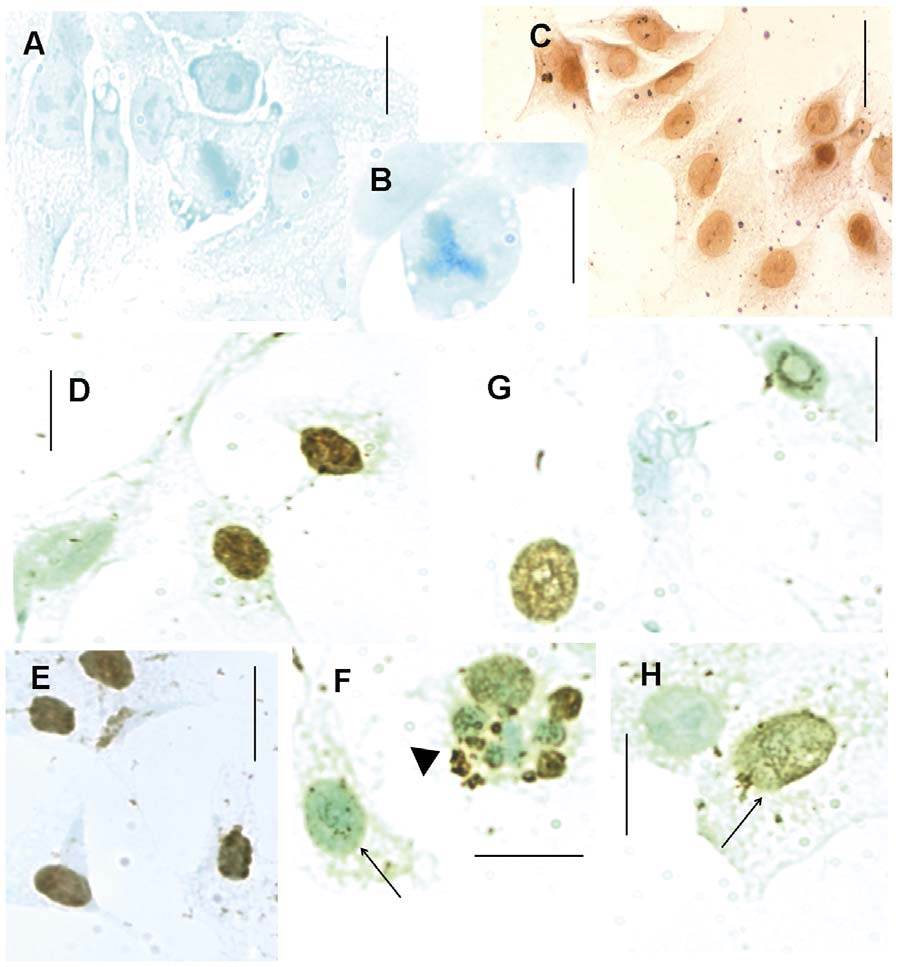
DNA fragmentation revealed in HeLa cells with the TUNEL assay. A, B. Negative control; C. Positive control. D-F. TSA-treated cells (arrowhead, cell death preceded by multinucleation); G, H. VPA-treated cells. Positive response intensities: strong (D, E), moderate (G, H (arrow)), weak (F, arrow). Scale bars, 25 µm.

No labeling was found in the absence of TdT (negative control) (Fig. 7 A, B), indicating that the positive immunocytochemical response was not caused by endogenous cell peroxidases. In addition, there was a very strong positive response in cells treated with DNase (positive control) (Fig. 7 C). Variations in the intensity of the positive response to the TUNEL assay (weak, moderate and strong) were detected in HeLa cells in the absence or presence of TSA or VPA treatment (Fig. 7 D–I, G, H; Table 6). DNA fragmentation was also detected in cells undergoing cell death preceded by multinucleation (Fig. 7 F).

While the positive response in the TUNEL assay increased with all doses of the 4 and 24-h TSA treatment used, only the 24 h VPA treatments induced a significant increase in this response (Table 6).

**Table 6:** TUNEL positivity in VPA-treated HeLa cells

Treatment			TUNEL positive response (%)			
time (h)	Drug	Concentration	Total	Strong	Moderate	Weak
1	VPA	zero	0.37^a^	0.00^a^	0.09^a^	0.28^a^
		0.05 mM	0.90^a^	0.00^a^	0.00^a^	0.90^a^
		0.5 mM	0.80^a^	0.00^a^	0.00^a^	0.80^a^
		1.0 mM	1.80^a^	0.00^a^	0.00^a^	1.80^a^
2	VPA	zero	2.35^a^	0.10^a^	0.30^a^	1.95^a^
		0.05 mM	3.60^a^	0.00^a^	0.10^a^	3.50^a^
		0.5 mM	4.40^a^	0.00^a^	0.60^a^	3.80^a^
		1.0 mM	4.00^a^	0.10^a^	0.60^a^	3.30^a^
4	VPA/TSA	zero	1.70^a^	0.00^a^	0.50^a^	1.20^a^
	VPA	0.05 mM	1.80^a^	0.00^a^	0.10^a^	1.70^a^
		0.5 mM	1.30^a^	0.10^a^	0.00^a^	1.20^a^
		1.0 mM	8.10^a^	3.50 a	1.70^a^	2.90^a^
	TSA	10 ng/mL	30.10^b^	12.60^b^	9.70^b^	7.80^a^
		20 ng/mL	35.90^b^	24.50^b^	6.30^b^	5.10^a^
		100 ng/mL	43.90^b^	18.95^b^	20.00^b^	4.95^a^
24	VPA/TSA	zero	5.90^a^	0.30 a	0.40^a^	5.20^a^
	VPA	0.05 mM	14.90 b	0.60 a	1.90 a	12.40^b^
		0.5 mM	21.50 b	1.10 a	1.20 a	19.20 b
		1.0 mM	36.00^b^	0.70 a	3.60 a	31.70 b
	TSA	10 ng/mL	11.00^a^	0.70 a	0.30 a	10.00^a^
		20 ng/mL	31.40 b	1.20 a	4.60 a	25.60 b
		100 ng/mL	60.90^b^	7.80^b^	13.60^b^	39.60^b^

b , VPA- or TSA-treated groups differ significantly from their respective controls (zero,

a ) at the P _0.05_ level (Mann-Whitney test); TSA, trichostatin A; VPA, valproic acid; n, 1000 cells per experimental condition.

## Discussion

The results indicate an induction of chromatin decondensation in VPA-treated HeLa cells as assessed by image analysis procedures. Image analysis cytometry similar to that employed here has allowed the identification of chromatin remodeling in several other cell types under different physiological or pathological conditions [23], [34], [35], [37]–[41]. Chromatin decondensation in HeLa cells was not a VPA-selective or direct effect, as it also occurred by HDAC inhibition in VPA-treated MCF-7 cells [7] and in TSA-treated HeLa cells [23], [24]. Chromatin decondensation in HeLa cells was verified at VPA exposure times as short as 1 h and at drug doses as low as 0. 25 mM. VPA-induced acetylation of histones H3 and H4 have been verified under these mild experimental conditions in MCF-7 cells [7].

The observed textural changes in the chromatin appeared under conditions in which several reports demonstrated that HDAC activity decreased and histone acetylation increased under treatment with VPA and TSA in HeLa cells [6], [16], [23]. Consequently, we found it unnecessary to repeat the previously reported biochemical experiment on HDAC activity and Western blot analysis in the present study. In addition, current studies in our laboratory involving image analysis of NIH 3T3 cells treated with VPA and TSA and a previous study on the human glioma NG97 cells, which is a cell line described to derive from a grade III astrocytoma [42], indicate similar textural changes in chromatin. The results for the NIH 3T3 cells are accompanied by increased histone H3 and H4 acetylation (demonstrated by Western blotting) and decreased HDAC activity.

It is well known that histone modification, specifically, histone acetylation and deacetylation, is a key factor in the regulation of gene expression, and that dynamic changes in gene expression can affect chromatin structure and its interaction with regulatory factors [7]. HDACis like VPA induce gene deregulation, leading, for instance, to the expression of specific genes [10], [43], [44] and to the induction of teratogenicity [22]. Because VPA elicits chromatin decondensation in HeLa cells, it most likely affects the expression of at least some of their genes. Indeed, treatment of HeLa cells with 3.0 mM VPA for 24 h, a condition that approaches that required for the induction of H4 hyperacetylation, has revealed deregulation of 6% of HeLa cell genes (1625), as assessed by DNA chip analyses [12]. In this case, more genes are up-regulated (1074) than down-regulated (551). In addition, more genes are deregulated by VPA than the knockdown of HDAC1, -2 and -3 [12]. Genes that were up-regulated more than 2-fold compared to those in the untreated or scrambled control groups were found to be related to the cell cycle (e.g., p21, HRAS-like suppressor 3 and cyclin D2), cell signaling (RAS guanyl releasing protein 2, transforming growth factor-alpha and glycoprotein hormones) and other functions (pyruvate dehydrogenase kinase 4 and ATPase class V, type IOD) [12]. Down-regulated genes were found to be related to importin β, Fas apoptotic inhibitory molecule and cyclin B1 [12]. In rat neurons treated with VPA or TSA, microarray analysis has revealed that 726 genes are up-regulated (particularly those involved in epileptogenesis), whereas 577 genes are down-regulated (particularly those responsible for the development of GABAergic inhibitory neurons) [45]. In this case, the acetylation of histones H3 and H4 is increased only in the promoters of the up-regulated genes [45].

It is worth mentioning that in the human breast cancer cell line MCF-7, the chromatin decondensation that is promoted in response to VPA-induced HDAC inhibition is accompanied by an increased sensitivity of DNA to nucleases and an increased association of DNA with intercalating agents that have been assumed to be modulated by the depletion of non-histone proteins involved in the maintenance of chromatin structure (SMC) [7]. A decrease in the expression of genes encoding the SMC proteins, SMC-associated proteins, DNA methyltransferase 1 and HP1 (heterochromatin protein 1), as determined by microarray analysis, has been ascribed to HDAC inhibition, rather than to a direct VPA effect [7]. In the present study, the role of depletion of such proteins on chromatin decondensation in HeLa cells cannot be ruled out.

In addition to histone hyperacetylation, DNA demethylation of specific genes is also triggered by VPA in HEK293 cells [13], [14], but not in FXS (fragile X syndrome) lymphoblastoid cell lines in which the FMR1 gene is silenced by DNA methylation [46] or in mouse embryos [47]. It should be emphasized that in contrast to histone acetylation, the induction of DNA demethylation by VPA can cause long-term consequences for the integrity of gene expression programming by reversing stable DNA methylation patterns in nondividing cells [13], [14]. HDAC inhibition by VPA and TSA has also been recently reported to induce histone methylation in rat cortical neurons and astrocytes in which these drugs up-regulate overall and gene-specific H3K4 di- and trimethylation [15] and to increase H3K4 methylation but decrease H3K9 methylation in mouse embryos [47]. There is no record of HDACi-promoted histone methylation or DNA demethylation in HeLa cells. Consequently, chromatin remodeling in HeLa cells, as found in this study, cannot be discussed in these terms. Certainly, further investigation is required, including image analysis of cells first treated with VPA and subsequently cultured in the absence of this drug, comparison of these results with data obtained on cells treated with 5-azacytidine and biochemical studies on this matter.

The increase with the VPA treatments in the number of nuclei containing subdiploid class Feulgen-DNA amounts, which is not accompanied by an increase in DNA fragmentation or in frequency of cell death images, probably indicates an increased apurinic acid solubilization during the acid hydrolysis phase of the Feulgen reaction. This increase in apurinic acid solubilization could be due to a decrease in resistance of the DNA to the hydrolytic process [48] because of the chromatin unraveling promoted by the HDACi. Indeed, VPA-induced chromatin decondensation in MCF-7 cells has been associated with an increased sensitivity of the DNA to nucleases and with an increased access of the DNA to intercalating agents [7]. Given these findings, the apparent decrease in the frequency of nuclei with the largest Feulgen-DNA values with the 0.05–1.0 mM VPA treatment for 1, 2, 4 and 24 h does not mean decreased cell proliferation, which is supported by the fact that mitotic indices are unaffected under these experimental conditions.

The significant decrease in mitotic indices revealed with the 5.0 mM VPA treatment for 48 h indicates that relatively long periods of treatment and/or high doses of this drug are required for an effect on HeLa cell proliferation, which confirms previous data for this cell line [16] and is also consistent with a report for leukemic cells [33]. Conversely, even under this antiproliferative condition, no effect was verified on the frequency of cell death, as determined by morphology in Feulgen-stained preparations, which was used for the calculation of cell death ratios in VPA-treated HeLa cells. However, DNA fragmentation, as identified by a positive response in the TUNEL assay, increased after a 24-h treatment with VPA and a ≥ 4-h treatment with TSA. This finding indicates that a longer VPA exposure time and/or a higher drug concentration is required for the DNA fragmentation pathways to proceed to chromatin condensation and the release of apoptotic bodies. As regards the TSA action, the much earlier induction of DNA fragmentation as found in the present study is in agreement with reports for apoptosis reported for HeLa and other tumor cell lines [28]–[30], [32], [49]. Additionally, DNA fragmentation could be facilitated by chromatin decondensation promoted by the HDAC inhibitors.

## Materials and Methods

### Cell Culture

HeLa cells at passage 176 were grown in Dulbecco's modified essential medium (DMEM) (Sigma®, St. Louis, USA) containing 10% fetal calf serum (FCS) (Cultilab®, Campinas, Brazil) and 50 µg/mL of garamicin (Sigma®) and incubated at 37°C in a 5% CO_2_ atmosphere. HeLa cells were originally acquired from the Instituto Adolfo Lutz (São Paulo, Brazil) at passage 126 which by turn had been commercially acquired from ATCC CCL-2 (Manassas, USA). The cells were seeded into 24-well plates (TPP, Trasadingen, Switzerland) containing round glass coverslips at a concentration of 5×10^4^ cells/mL in complete medium for 24 h. Next they were treated with VPA (Sigma®) dissolved in PBS and diluted in the DMEM medium supplemented with 1% FCS at doses of 0.05, 0.5 or 1.0 mM for varying times (1, 2, 4 and 24 h). Only to evaluate mitotic indices, nuclear abnormalities and cell death ratios, cells treated with VPA at the dose of 5.0 mM for 48 h was also analyzed. VPA at concentrations of 0.3–1.0 mM acts as a potent inhibitor of HDAC activity [5]. TSA (Sigma®) dissolved in DMSO (Labsynth, Diadema, Brazil) and diluted in medium containing 1% FCS at the dose of 100 ng/mL for 24 h (image analysis) and at the doses of 10, 20 and 100 ng/mL for 4 and 24 h (TUNEL assay) was used as a positive control of pan-HDACI activity [23], [24]. Controls in the absence of the drugs were also used.

### Cell Fixation and DNA Topochemistry

The cells adhered to round glass coverslips were fixed in a mixture of absolute ethanol-glacial acetic acid (3∶1, v/v) for 1 min, rinsed in 70% ethanol and air dried at room temperature. Another set of cell preparations was fixed in 4% paraformaldehyde for 15 min, rinsed in phosphate saline buffer and air dried.

The cells fixed in acetic ethanol were used for DNA topochemistry and image analysis, and for the establishment of mitotic indices and apoptotic ratios. They were subjected to the Feulgen reaction with hydrolysis carried out in 4 M HCl for 60 min at 25°C. The hydrolyzed material was treated with Schiff reagent for 40 min, rinsed three times (5 min each) in sulfurous water and once in distilled water, air dried, cleared in xylene and mounted in Canada balsam (n_D_ = 1.54). Acetic ethanol-fixed smears of human lymphocytes were subjected to the Feulgen reaction under hydrolysis conditions which enabled maximal depurination (4 M HCl for 90 min at 25°C). These cells were used as a control for 2 C ploidy.

The cells fixed in paraformaldehyde were used for the terminal deoxynucleotidyl transferase (TdT)-mediated dUTP nick 3′-end DNA labeling (TUNEL) assay.

### Scanning Microspectrophotometry Image Analysis

Images of the Feulgen-stained nuclei were obtained with a Zeiss automatic scanning microspectrophotometer (Carl Zeiss, Oberkochen, Germany) interfaced to a personal computer. The operating conditions used were as follows: Planapo objective 63/0.90; optovar 2.0; measuring diaphragm diameter, 0.25 mm; field diaphragm diameter, 0.20 mm; LD-Epiplan 16/0.30 condenser; scanning spot of 0.5 µm x 0.5 µm; halogen 100-W/12-V lamp; stabilized electronic power supply, Zeiss light modulator; λ = 565 nm obtained with a Schott monochromator filter ruler; R-928 photomultiplier; and a Pentium II microcomputer. Grid points (individual measuring points) showing absorbances no higher than 0.020 were considered to be background and were automatically removed from the nuclear image. The cutoff point of 0.100 was selected to evaluate the areas covered by condensed chromatin in HeLa cells after a preliminary test done for untreated controls. Two hundred nuclei were chosen at random and measured individually for each experimental condition.

The image analysis parameters pertinent to this investigation were as follows: A_T_, total integrated absorbance  =  nuclear Feulgen-DNA values or IOD (integrated optical density) in arbitrary units; A_C_, integrated absorbance over the preselected cutoff (”condensed” chromatin Feulgen-DNA values); A_C_ %, “condensed” chromatin Feulgen-DNA values relative to the nuclear (whole chromatin) Feulgen-DNA values; S_T_, nuclear absorbing area in µm^2^; S_C_, area in µm^2^ covered with stained chromatin showing absorbance above the selected cutoff point; S_C_ %, area covered with “condensed” chromatin relative to the nuclear area; AAR  =  (A_C_/S_C_)/(A_T_/S_T_), average absorption ratio, a dimensionless parameter that expresses how many times the average absorbance of the “condensed” chromatin exceeds that of the entire nucleus [50]. A scatter diagram relating AAR to S_C_% was plotted as previously proposed [34], [51]. This diagram allows for the discrimination of the position of points that correspond to specific nuclear phenotypes [34], [35], [51].

### Mitotic Abnormalities, Micronucleation, Mitotic Indices and Cell Death Ratios

The frequency of abnormal metaphases, lagging chromosomes, chromosome bridging, giant nuclei, micronucleation and mitotic indices were estimated in Feulgen-stained preparations.

Apoptotic cells and cells identified as those suffering “cell death preceded by multinucleation” (denomination recommended in substitution to mitotic catastrophe) [52] were also evaluated in the preparations subjected to the Feulgen reaction. Apoptotic cells were considered here as cells presenting extremely condensed chromatin or apoptotic bodies. Approximately 2000 cells per preparation were examined in all cases.

### TUNEL Assay

TUNEL analysis was performed following the manufacturer's instructions (Roche, Mannheim, Germany). Endogenous peroxidase was blocked with 3% H_2_O_2_ in absolute methanol for 10 min at room temperature. Some preparations were processed by omitting TdT (negative control) or by including a DNase treatment before TdT addition (positive control). Counterstaining was performed in a methyl green (Merck, Darmstadt, Germany) solution. Nuclei with brown-stained spots indicated TUNEL positive cells. The intensities of positive responses in the TUNEL assay were visually ranked as weak, moderate or strong. This classification was supported by a quantitative image analysis approach, which was performed by defining values of nuclear relative areas covered by the “dark” brown spots that resulted from the immunocytochemical assay. Image analysis was conducted with Kontron KS400 version 3 software and Zeiss Axiophot 2/Kontron equipment (Oberkochen/Munich, Germany), using a Neofluar objective 40/0.75, an optovar factor of 1.4 and a Zeiss AxioCam HRc color video camera. After the area for the whole nucleus was computed, the definition of the image area covered by the dark spots was obtained in this system by fixing the low and high threshold range levels to 60 and 100 units, respectively. While maintaining these parameters, nuclei with a strong response in the TUNEL assay were defined as those that contained dark spots over ≥ 50% of their area. Nuclei with moderate and weak responses contained dark spots over 5–49% and <5% of their areas, respectively. The use of mean gray values as a quantitative parameter for discrimination of the TUNEL response did not appear adequate because the reaction observed in this assay was not found to conform to the Beer-Lambert law. The TUNEL assay response was determined for 1000 cells under each experimental condition. Three coverslips for each experimental condition were examined.

### Statistics

Calculations and statistical analyses were performed using Minitab 12™ software (State College, PA, USA). Statistical significance was calculated by the two-sided Student t-test when the samples showed a normal distribution. The Mann-Whitney test was performed to assess the statistical significance when the samples did not show a normal distribution. P_<0.05_ was considered the critical level for rejection of the null hypothesis.

## Supporting Information

Figure S1 **Frequency histograms of Feulgen-DNA values in HeLa cells treated with VPA for 1 h.** n, 200. Human lymphocytes were used as a reference for class 2 C Feulgen-DNA.
(TIF)

Figure S2 **Frequency histograms of Feulgen-DNA values in HeLa cells treated with VPA for 2 h.** n, 200. For the reference on human lymphocyte, see Fig. S1.
(TIFF)

Figure S3 **Frequency histograms of Feulgen-DNA values in HeLa cells treated with VPA for 4 h.** n, 200. For the reference on human lymphocyte, see Fig. S1.
(TIFF)

Figure S4 **Frequency histograms of Feulgen-DNA values in HeLa cells treated with VPA for 24 h.** n, 200. For the reference on human lymphocyte, see Fig. S1.
(TIFF)

Table S1 **Mitotic index, nuclear abnormalities and cell death ratios in Feulgen-stained VPA-treated HeLa cells.**
(DOC)

## References

[1] Phiel CJ, Zhang F, Huang EY, Guenther MG, Lazar MA (2001). Histone deacetylase is a direct target of valproic acid, a potent anticonvulsant, mood stabilizer, and teratogene.. J Biol Chem.

[2] Frazee LA, Foraker KC (2008). Use of intravenous valproic acid for acute migraine.. Ann Pharmacother.

[3] Silva MFB, Aires CCP, Luis PBM, Ruiter JPN, IJist L (2008). Valproic acid metabolism and its effects on mitochondrial fatty acid oxidation: A review.. J Inherit Metab Dis.

[4] Perucca E (2002). Overtreatment in epilepsy: adverse consequences and mechanisms.. Epilepsy Res.

[5] Göttlicher M, Minucci S, Zhu P, Kramer OH, Schimpf A (2001). Valproic acid defines a novel class of HDAC inhibitors inducing differentiation of transformed cells.. Embo J.

[6] Eyal S, Yagen B, Sobol E, Altschuler Y, Shmuel M (2004). The activity of antiepileptic drugs as histone deacetylase inhibitors.. Epilepsia.

[7] Marchion DC, Bicaku E, Daud AI, Sullivan DM, Munster PN (2005). Valproic acid alters chromatin structure by regulation of chromatin modulation proteins.. Cancer Res.

[8] Kortenhorst MSQ, Isharwal S, van Diest P, Chowdury WH, Marlow C (2009). Valproic acid causes dose- and time-dependent changes in nuclear structure in prostate cancer cells in vitro and in vivo.. Mol Cancer Ther.

[9] Fortson WS, Kayarthodi S, Fujimura Y, Xu HL, Matthews R (2011). Histone deacetylase inhibitors, valproic acid and trichostatin-A induce apoptosis and affect acetylation status of p53 in ERG-positive prostate cancer cells.. Int J Oncol.

[10] Xu XXS, Wang L, Abrams J, Wang G (2011). Histone acetylases (HDACs) in XPC gene silencing and bladder cancer.. J Hematol Oncol.

[11] Khan N, Jeffers M, Kumar S, Hackett C, Boldog F (2008). Determination of the class and isoform selectivity of small-molecule histone deacetylase inhibitors.. Biochem J.

[12] Dejligbjerg M, Grauslund M, Litman T, Collins L, Qian X (2008). Differential effects of class I isoform histone deacetylase depletion and enzymatic inhibition by belinostat or valproic acid in HeLa cells.. Mol Cancer.

[13] Detich N, Bovenzi V, Szyf M (2003). Valproate induces replication-independent active DNA demethylation.. J Biol Chem.

[14] Milutinovic S, D'Alessio AC, Detich N, Szyf M (2007). Valproate induces widespread epigenetic reprogramming which involves demethylation of specific genes.. Carcinogenesis.

[15] Marinova Z, Leng Y, Leeds P, Chuang DM (2011). Histone deacetylase inhibition alters histone methylation associated with heat shock protein 70 promoter modifications in astrocytes and neurons.. Neuropharmacol.

[16] Sami S, Höti N, Xu HM, Shen Z, Huang X (2008). Valproic acid inhibits the growth of cervical cancer both *in vitro* and *in vivo*.. J Biochem.

[17] Gotfryd K, Hansen M, Kawa A, Ellerbeck U, Nau H (2011). The teratogenic potenties of valproic acid derivatives and their effects on biological end-points are related to change in histone deacetylase and Erk1/2 activities.. Basic & Clin Pharm Toxicol.

[18] Li XN, Shu Q, Su JMF, Perlaky L, Blaney SM (2005). Valproic acid induces growth arrest, apoptosis, and senescence in medulloblastomas by increasing histone hyperacetylation and regulating expression of p21Cip1, CDK4, and CMYC.. Mol Cancer Therap.

[19] Atmaca A, Al-Batran SE, Maurer A, Neumann A, Heinzel T (2007). Valproic acid (VPA) in patients with refractory advanced cancer: a dose escalating phase I clinical trial.. Br J Cancer.

[20] Soriano AO, Yang H, Faderl S, Estrov Z, Giles F (2007). Safety and clinical activity of the combination of 5-azacytidine, valproic acid, and all-trans retinoic acid in acute myeloid leukemia and myelodisplastic syndrome.. Blood.

[21] Braiteh F, Soriano AO, Garcia-Manero G, Hong D, Johnson MM (2008). Phase I study of epigenetic modulation with 5-azacytidine and valproic acid in patients with advanced cancers.. Clin Cancer Res.

[22] Jergil M, Forsberg M, Salter H, Stockling K, Gustafson AL (2011). Short-time gene expression response to valproic acid analogs in mouse embryonic stem cells.. Toxic Sci.

[23] Tóth KF, Knoch TA, Wachsmuth M, Frank-Stohr M, Stohr M (2004). Trichostatin A-induced histone acetylation causes decondensation of interphase chromatin.. J Cell Sci.

[24] Rao J, Bliattacharya D, Banerjee B, Sarin A, Shivashankar GV (2007). Trichostatin-A induces differential changes in histone protein dynamics and expression in HeLa cells.. Biochem Biophys Res Commun.

[25] Shin HJ, Baek KH, Jeon AH, Kim SJ, Jang KL (2003). Inhibition of histone deacetylase activity increases chromosomal instability by the aberrant regulation of mitotic checkpoint activation.. Oncogene.

[26] Bolden JE, Peart MJ, Johnstone RW (2006). Anticancer activities of histone deacetylase inhibitors.. Nature Rev Drug Discov.

[27] Xu WS, Parmigiani RB, Marks PA (2007). Histone deacetylase inhibitors: molecular mechanisms of action.. Oncogene.

[28] Gan YH, Wang J, Coselli J, Wang XL (2008). Synergistic induction of apoptosis by HMG-CoA reductase inhibitor and histone deacetylases inhibitor in HeLa cells.. Biochem Biophys Res Commun.

[29] Hayakawa F, Abe A, Kitabayashi I, Pandolfi PP, Naoe T (2008). Acetylation of PML is involved in histone deacetylase inhibitor-mediator apoptosis.. J Biol Chem.

[30] Liu PY, Chan JYH, Lin HC, Wang SL, Liu ST (2008). Modulation of cyclin-dependent kinase inhibitor p21^WAF1/Cip1^ gene by Zac1 through the antagonistic regulators p53 and histone deacetylase 1 in HeLa cells.. Mol Cancer Res.

[31] Frew AJ, Johnstone RW, Bolden JE (2009). Enhancing the apoptotic and therapeutic effects of HDAC inhibitors.. Cancer Lett.

[32] Noh EJ, Lim DS, Jeong GJ, Lee JS (2009). An HDAC inhibitor, trichostatin A, induces a delay at G_2_/M transition, slippage of spindle checkpoint, and cell death in a transcription-dependent manner.. Biochem Biophys Res Commun.

[33] Elknerova K, Myslivcova D, Lacinova Z, Marinov I, Uherkova L (2011). Epigenetic modulation of gene expression of human leukemia cell lines – induction of cell death and senescence.. Neoplasma.

[34] Vidal BC (1984). Polyploidy and nuclear phenotypes in salivary glands of the rat.. Biol Cell.

[35] Mello MLS, Aldrovani M, Moraes AS, Guaraldo AMA, Vidal BC (2009). DNA content, chromatin supraorganization, nuclear glycoproteins and RNA amounts in hepatocytes of mice expressing insulin-dependent diabetes.. Micron.

[36] Johnson RT, Mullinger AM, Downes CS, Prescott DM (1978). Human minisegregant cells.. Methods in Cell Biology, Vol 20.

[37] Mello MLS, Russo J (1990). Image analysis of Feulgen-stained c-H-*ras*-transformed NIH/3T3 cells.. Biochem Cell Biol.

[38] Mello MLS, Contente S, Vidal BC, Planding W, Schenck U (1995). Modulation of *ras* transformation affecting chromatin supraorganization as assessed by image analysis.. Expt Cell Res.

[39] Vidal BC, Russo J, Mello MLS (1998). DNA content and chromatin texture of benzo[a]pyrene-transformed human breast epithelial cells as assessed by image analysis.. Expt Cell Res.

[40] Moraes AS, Vidal BC, Guaraldo AM, Mello MLS (2005). Chromatin supraorganization and extensibility following starvation and refeeding.. Cytometry Part A.

[41] Moraes AS, Guaraldo AM, Mello MLS (2007). Chromatin supraorganization and extensibility in mouse hepatocytes with development and aging.. Cytometry Part A.

[42] Machado CML, Ikemori RY, Zorzeto TQ, Nogueira ACMA, Barbosa SDS (2008). Characterization of cells recovered from the xenotransplanted NG97 human-derived glioma cell line subcultured in a long-term in vitro.. BMC Cancer.

[43] Lagace DC, McLeod RS, Nachtigal MW (2004). Valproic acid inhibits leptin secretion and reduces leptin messenger ribonucleic acid levels in adipocytes.. Endocrinology.

[44] Qiao LP, Schaack J, Shao JH (2006). Suppression of adiponectin gene expression by histone deacetylase inhibitor valproic acid.. Endocrinology.

[45] Fukuchi M, Nii T, Ishimaru N, Minamino A, Hara D (2009). Valproic acid induces up- or down-regulation of gene expression responsible for the neuronal excitation and inhibition in rat cortical neurons through its epigenetic actions.. Neurosci Res.

[46] Tabolacci E, De Pascalis I, Accadia M, Terracciano A, Moscato U (2008). Modest reactivation of the mutant FMR1 gene by valproic acid is accompanied by histone modifications but not DNA demethylation.. Pharmacogenet Genomics.

[47] Tung EWY, Winn LM (2010). Epigenetic modifications in valproic acid-induced teratogenesis.. Toxicol Appl Pharmacol.

[48] Mello MLS, Vidal BC (1980). Acid lability of deoxyribonucleic acids of some polytene chromosome regions of *Rhynchosciara americana*.. Chromosoma.

[49] Platta CS, Greenblatt DY, Vaccaro A, Kunni-malaiyaan M, Chen H (2007). Trichostatin A induces morphologic differentiation and inhibits growth in small cell lung cancer cells.. J Surg Res.

[50] Vidal BC, Schlüter G, Moore GW (1973). Cell nucleus pattern recognition: influence of staining.. Acta Cytol.

[51] Mello MLS, Vidal BC, Planding W, Schenck U (1994). Image analysis: video system adequacy for the assortment of nuclear phenotypes based on chromatin texture evaluation.. Acta Histochem Cytochem.

[52] Kroemer G, Galluzzi L, Vandenabeele P, Abrams J, Alnemri ES (2009). Classification of cell death: recommendations of the Nomenclature Committee on Cell Death 2009.. Cell Death Differ.

