# Supplementary figures and images for: Chromatin Remodeling, Cell Proliferation and Cell Death in Valproic Acid-Treated HeLa Cells
Source: PLoS One. 2011 Dec 19;6(12):e29144. doi: 10.1371/journal.pone.0029144 (PMC3242782; doi:10.1371/journal.pone.0029144)

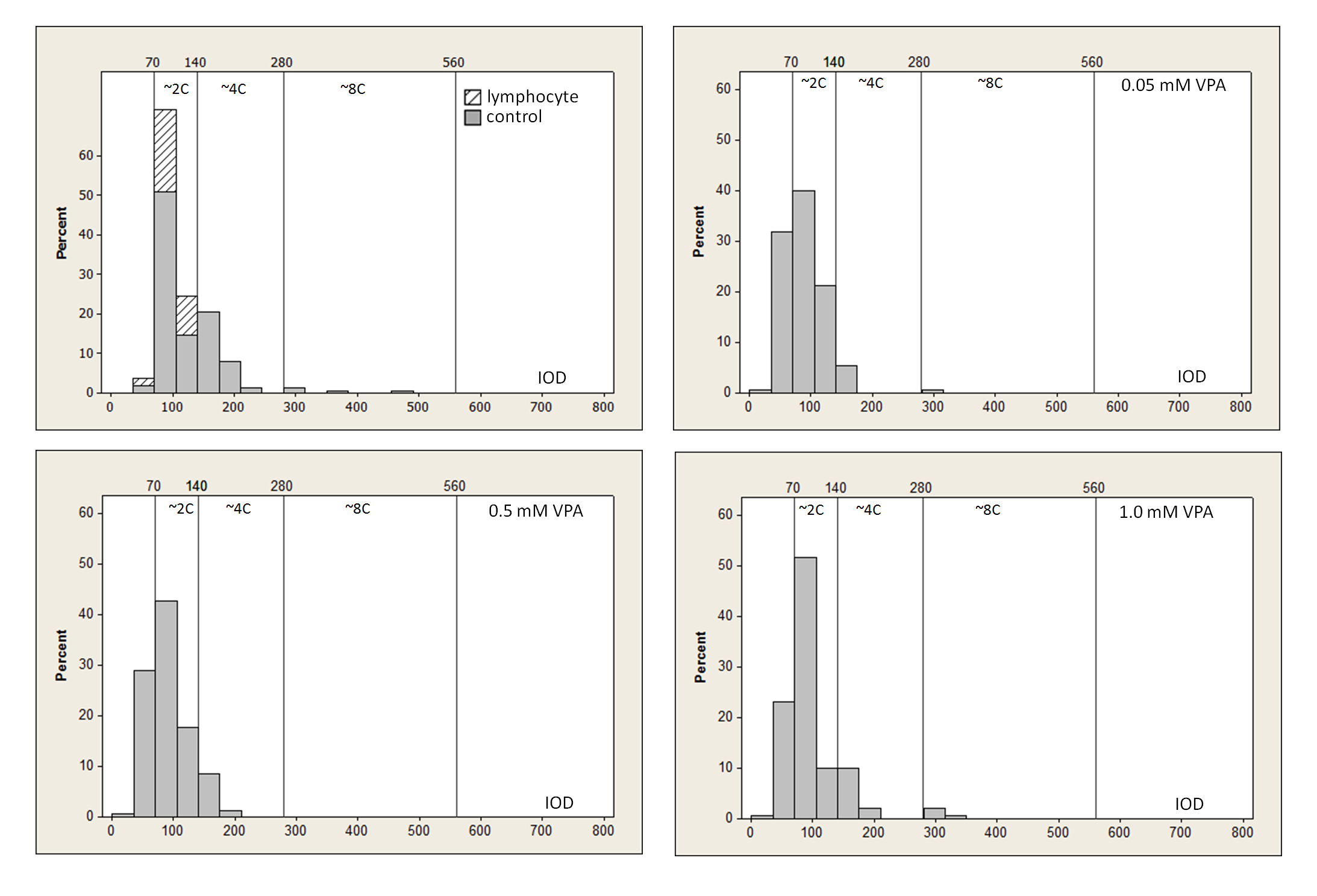

Supplement: Figure S1 — Frequency histograms of Feulgen-DNA values in HeLa cells treated with VPA for 1 h. n, 200. Human lymphocytes were used as a reference for class 2 C Feulgen-DNA. (TIF) [file pone.0029144.s001.tif]

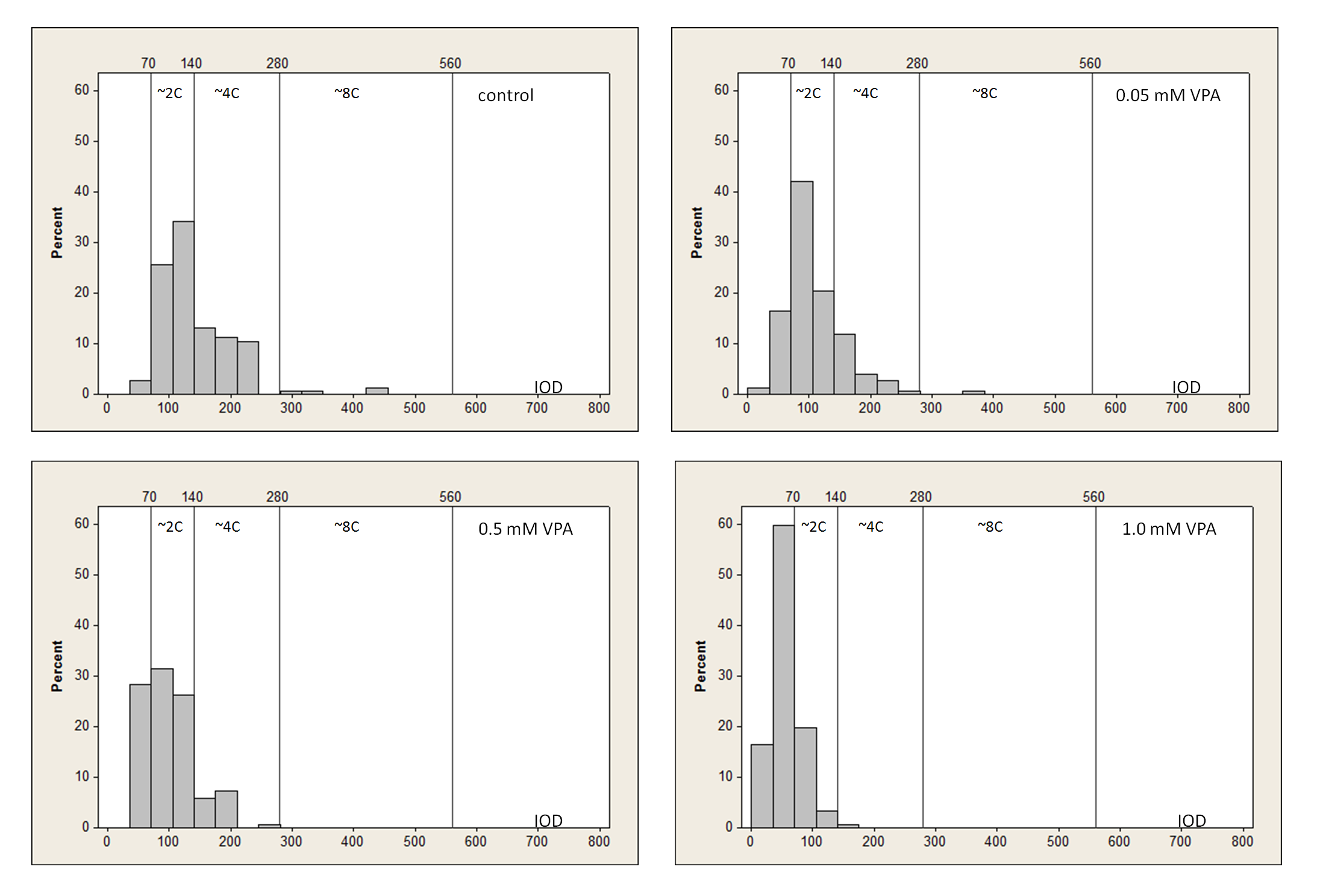

Supplement: Figure S2 — Frequency histograms of Feulgen-DNA values in HeLa cells treated with VPA for 2 h. n, 200. For the reference on human lymphocyte, see Fig. S1. (TIFF) [file pone.0029144.s002.tif]

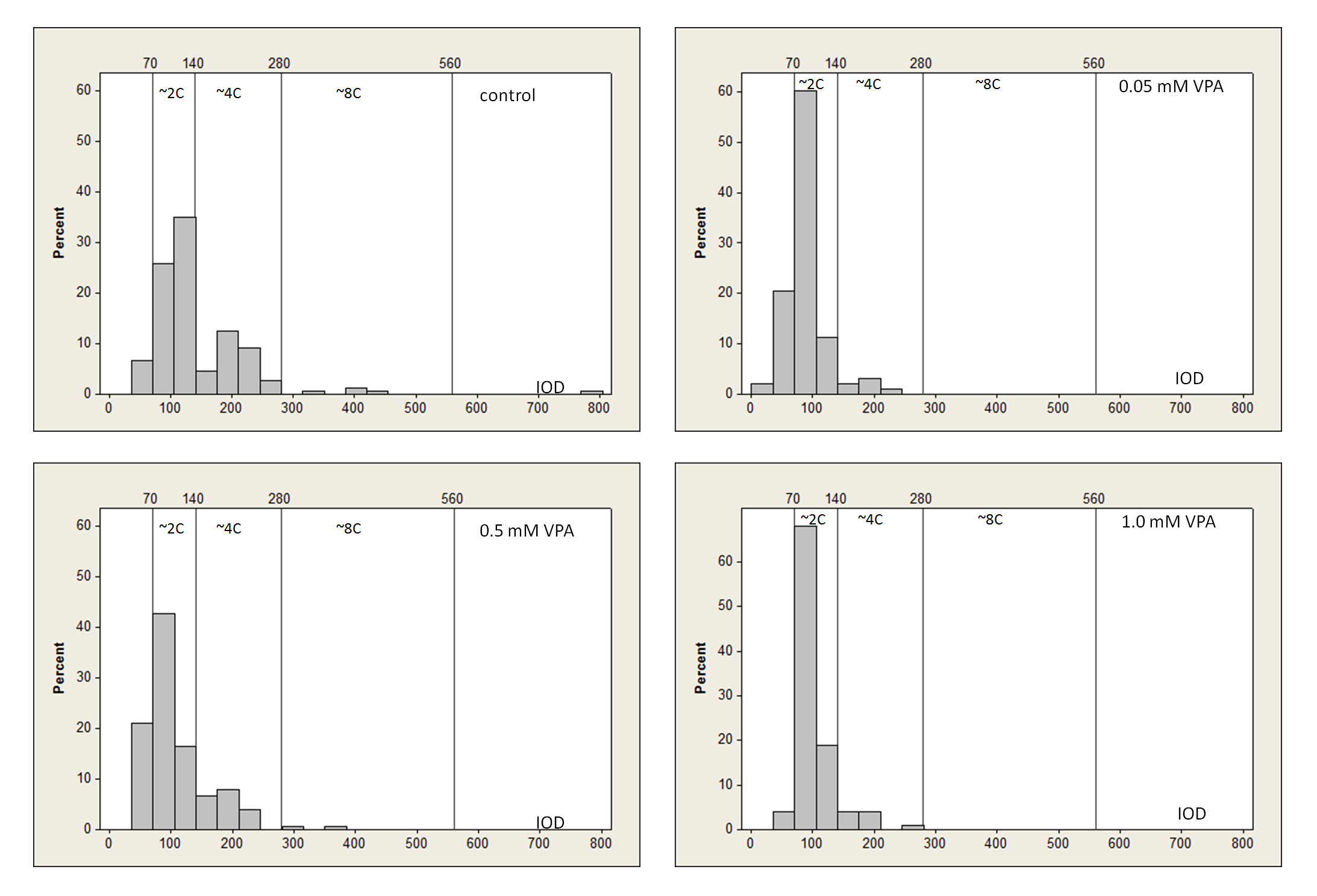

Supplement: Figure S3 — Frequency histograms of Feulgen-DNA values in HeLa cells treated with VPA for 4 h. n, 200. For the reference on human lymphocyte, see Fig. S1. (TIFF) [file pone.0029144.s003.tif]

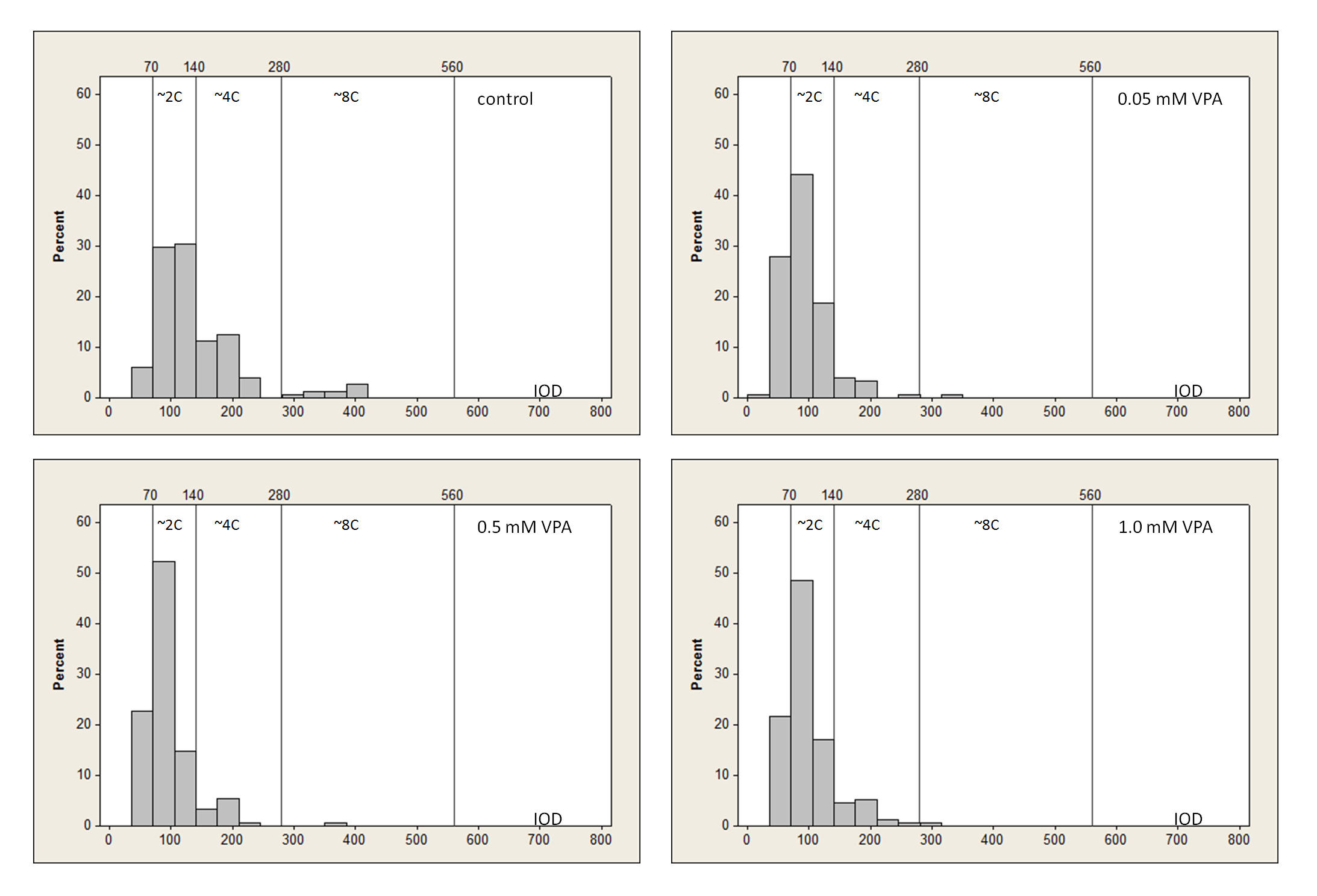

Supplement: Figure S4 — Frequency histograms of Feulgen-DNA values in HeLa cells treated with VPA for 24 h. n, 200. For the reference on human lymphocyte, see Fig. S1. (TIFF) [file pone.0029144.s004.tif]
