# Supplementary material for: Chromatin Remodeling, Cell Proliferation and Cell Death in Valproic Acid-Treated HeLa Cells
Source: PLoS One. 2011 Dec 19;6(12):e29144. doi: 10.1371/journal.pone.0029144 (PMC3242782; doi:10.1371/journal.pone.0029144)
Supplement: Table S1 — Mitotic index, nuclear abnormalities and cell death ratios in Feulgen-stained VPA-treated HeLa cells. (DOC) [file pone.0029144.s005.doc]

**Table S1**. Mitotic index, nuclear abnormalities and cell death ratios in Feulgen-stained VPA-treated HeLa cells

| Treatment | | Mitotic index (%) | | | Abnormal mitosis (%) | | | Giant nuclei (%) | | | Micronuclei (%) | | | Apoptotic ratio (%) | | | CDPM ratio (%) | | |
| --- | --- | --- | --- | --- | --- | --- | --- | --- | --- | --- | --- | --- | --- | --- | --- | --- | --- | --- | --- |
| Time (h) | VPA (mM) | X | S | Md | X | S | Md | X | S | Md | X | S | Md | X | S | Md | X | S | Md |
| 1 | zero | 2.97 | 0.82 | 2.63 | 60.95 | 7.29 | 64.00 | 0.23 | 0.15 | 0.20 | 2.63 | 0.82 | 2.63 | 0.20 | 0.17 | 0.15 | 0.23 | 0.20 | 0.30 |
|  | 0.05 | 1.99 | 0.62 | 2.17 | 55.48 | 3.31 | 56.25 | 0.22 | 0.08 | 0.20 | 3.51 | 2.83 | 2.89 | 0.07 | 0.08 | 0.05 | 0.35 | 0.27 | 0.30 |
|  | 0.5 | 2.34 | 0.42 | 2.28 | 49.70 | 23.30 | 60.60 | 0.20 | 0.13 | 0.25 | 2.30 | 0.73 | 2.13 | 0.21 | 0.12 | 0.20 | 0.25 | 0.22 | 0.15 |
|  | 1.0 | 2.85 | 1.91 | 2.60 | 61.68 | 13.65 | 65.06 | 0.20 | 0.05 | 0.20 | 3.53 | 2.39 | 3.05 | 0.30 | 0.30 | 0.15 | 0.33 | 0.24 | 0.44 |
| 2 | zero | 2.67 | 0.96 | 2.44 | 58.84 | 10.70 | 60.87 | 0.22 | 0.16 | 0.15 | 3.34 | 1.17 | 3.63 | 0.26 | 0.12 | 0.25 | 0.20 | 0.09 | 0.20 |
|  | 0.05 | 2.87 | 1.43 | 2.28 | 52.98 | 17.04 | 61.97 | 0.20 | 0.05 | 0.20 | 2.98 | 1.97 | 2.00 | 0.20 | 0.13 | 0.15 | 0.12 | 0.08 | 0.10 |
|  | 0.5 | 2.94 | 1.57 | 3.58 | 56.89 | 2.82 | 57.41 | 0.08 | 0.03 | 0.10 | 2.91 | 1.63 | 2.69 | 0.33 | 0.14 | 0.30 | 0.18 | 0.14 | 0.10 |
|  | 1.0 | 2.08 | 0.59 | 1.91 | 45.20 | 12.11 | 50.00 | 0.13 | 0.03 | 0.15 | 3.75 | 2.58 | 4.33 | 0.38 | 0.31 | 0.20 | 0.25 | 0.13 | 0.29 |
| 4 | zero | 2.45 | 0.74 | 2.09 | 55.14 | 10.43 | 50.00 | 0.10 | 0.10 | 0.10 | 3.13 | 1.15 | 3.19 | 0.32 | 0.26 | 0.25 | 0.12 | 0.11 | 0.05 |
|  | 0.05 | 1.90 | 0.56 | 2.20 | 66.48 | 15.22 | 69.44 | 0.12 | 0.10 | 0.15 | 4.47 | 1.36 | 4.02 | 0.21 | 0.04 | 0.20 | 0.07 | 0.08 | 0.05 |
|  | 0.5 | 2.71 | 1.03 | 2.90 | 31.10 | 11.44 | 30.43 | 0.20 | 0.13 | 0.15 | 2.43 | 0.68 | 2.81 | 0.12 | 0.08 | 0.10 | 0.23 | 0.23 | 0.10 |
|  | 1.0 | 1.91 | 0.08 | 1.92 | 58.89 | 13.24 | 54.84 | 0.24 | 0.14 | 0.30 | 5.37 | 2.47 | 5.18 | 0.19 | 0.11 | 0.20 | 0.17 | 0.19 | 0.08 |
| 24 | zero | 2.74 | 0.26 | 2.74 | 53.69 | 7.15 | 52.50 | 0.15 | 0.09 | 0.10 | 3.26 | 1.33 | 2.58 | 0.54 | 0.39 | 0.45 | 0.20 | 0.04 | 0.20 |
|  | 0.05 | 2.85 | 0.79 | 2.98 | 56.76 | 8.55 | 55.17 | 0.32 | 0.10 | 0.35 | 3.46 | 1.64 | 3.45 | 0.38 | 0.13 | 0.40 | 0.18 | 0.03 | 0.20 |
|  | 0.5 | 2.23 | 0.23 | 2.19 | 49.17 | 1.44 | 50.00 | 0.38 | 0.11 | 0.45 | 2.78 | 0.42 | 2.54 | 0.53 | 0.13 | 0.55 | 0.27 | 0.16 | 0.20 |
|  | 1.0 | 2.54 | 0.50 | 2.69 | 47.46 | 11.71 | 42.22 | 0.15 | 0.05 | 0.15 | 2.36 | 1.03 | 1.85 | 0.43 | 0.18 | 0.45 | 0.10 | 0.09 | 0.15 |
| 48 | zero | 2.42 | 0.78 | 2.65^a^ | 42.45 | 9.37 | 40.45 | 0.50 | 0.08 | 0.50^a^ | 0.14 | 0.09 | 0.17 | 0.51 | 0.27 | 0.55 | 0.25 | 0.26 | 0.20 |
|  | 5.0 | 0.50 | 0.26 | 0.45^b^ | 70.00 | 44.70 | 100. | 0.18 | 0.11 | 0.15^b^ | 0.08 | 0.07 | 0.10 | 0.55 | 0.31 | 0.45 | 0.20 | 0.14 | 0.20 |

Superscript letters a and b in the Md columns of the mitotic index and of the giant nuclei indicate that the VPA group differs significantly from respective control at the P _0.05_ level (Mann-Whitney test); CDPM, cell death preceded by multinucleation; Md, median; S, standard deviation; VPA, valproic acid; X, arithmetic mean; n = 2000 cells per experimental condition; number of preparations per experimental condition = 3
